# Prevalence and influencing factors of oral frailty in older adults with diabetes: a systematic review and meta-analysis

**DOI:** 10.3389/fpubh.2025.1702509

**Published:** 2025-11-28

**Authors:** Xiaoyu Wang, Xin Wang, Wanying Zhao

**Affiliations:** School of Nursing and Health, Zhengzhou University, Zhengzhou, China

**Keywords:** oral frailty, diabetes, older adults, prevalence, influencing factors, meta-analysis

## Abstract

**Objective:**

To systematically evaluate the prevalence and influencing factors of oral frailty in older adults with diabetes.

**Methods:**

We systematically searched for studies on the prevalence and influencing factors of oral frailty in older adults with diabetes across databases including CNKI, Wanfang Database, VIP Database, China Biology Medicine Disc (CBM), PubMed, Embase, Web of Science, CINAHL, and the Cochrane Library, from database inception to August 7, 2025.

**Results:**

Nine studies were ultimately included, involving a total of 2,395 patients. The meta-analysis showed that the prevalence of oral frailty in older adults with diabetes was 52% (95% CI: 0.43–0.61, *I*^2^ = 95.15%, *P* < 0.01). Influencing factors included age [odds ratio (OR) = 3.91, 95% CI: 2.13–5.68], smoking (OR = 4.64, 95% CI: 1.95–7.33), polypharmacy (OR = 8.70, 95% CI: 3.26–14.14), duration of diabetes (OR = 3.29, 95% CI: 1.55–5.04), glycated hemoglobin (HbA1c) level (OR = 3.61, 95% CI: 1.47–5.74), number of remaining teeth (OR = 11.84, 95% CI: 4.47–19.22), and oral health-related self-efficacy (OR = 0.18, 95% CI: 0.10–0.27).

**Conclusion:**

The prevalence of oral frailty is high among older adults with diabetes and is influenced by multiple factors. Healthcare providers should incorporate the assessment of oral frailty into routine dynamic monitoring for these patients. Based on individual conditions and clinical contexts, personalized, comprehensive, and early intervention plans should be developed through a multidisciplinary approach to prevent or delay the progression of oral frailty.

**Systematic review registration:**

https://www.crd.york.ac.uk/PROSPERO/view/CRD420251119100, identifier CRD420251119100.

## 1 Introduction

In China, the number of adults aged 20–79 years with diabetes has reached 148 million in 2024 is projected to increase to 168 million by 2050, and the prevalence of diabetes among older adults is 30.2% (1, 2). China has the largest diabetic population globally and the second highest total healthcare expenditure related to diabetes worldwide (1), highlighting its significance as a major public health challenge.

Oral frailty refers to the progressive decline in oral health associated with aging, encompassing reduction in tooth number, impaired oral hygiene and oral function, reduced attention to oral health, diminished physical and cognitive reserves, and difficulties in eating, ultimately contributing to overall functional deterioration (3). The “Healthy China 2030” blueprint has explicitly emphasized the importance of oral health and set key goals for improving oral hygiene and preventing oral disease (4). Diabetes and its complications are established risk factors for various oral health disorders, such as periodontitis, oral infections, halitosis, salivary dysfunction, dental caries, and tooth loss (5, 6). Therefore, oral health issues among diabetic patients—particularly older adults—require urgent attention. Given that many factors associated with oral frailty are reversible (7), early assessment of oral frailty in older diabetic patients can facilitate timely interventions and play a crucial role in maintaining their oral health.

However, current evidence on the prevalence and influencing factors of oral frailty in this population remains inconclusive. Most studies are small-scale, cross-sectional, which limiting the generalizability of their findings. Thus, this systematic review and meta-analysis aims to synthesize existing evidence to determine the prevalence and influencing factors of oral frailty among older adults with diabetes. The results may provide a scientific evidence for nurses to identify high-risk individuals early and develop tailored oral healthcare strategies to improve patient outcomes, thereby informing public health strategies for aging populations with diabetes.

## 2 Methods

This systematic review was reported in accordance with the Preferred Reported Items for Systematic Reviews and Meta-Analyses (PRISMA) guidelines. The study was registered at the International Register of Systematic Reviews (PROSPERO) with registration number CRD420251119100.

### 2.1 Search strategy

Two researchers independently conducted an electronic database search of Chinese and English databases, including PubMed, Embase, Cochrane Library, Web of Science, CINAHL, CNKI, WANFANG, VIP Database, and SinoMed. The search was performed from inception until August 7, 2025, using a combination of Medical Subject Headings terms and free-text words. Moreover, relevant citations were manually retrieved from the reference lists of included studies and other published meta-analyses. The search strategy included terms related to “oral frailty” and “Diabetes” and “older adult” (See Supplementary material S1 for full search strategy).

### 2.2 Inclusion and exclusion criteria

The criteria for inclusion of a study in the systematic review were as follows: (1) participants were older adults with diabetes (age ≥60 years, type 1/type 2 diabetes); (2) study designs were cross-sectional, cohort, or case-control; (3) clear diagnostic criteria for oral frailty; and (4) reporting data on the prevalence of oral frailty and influencing factors, with multivariate analysis results provided.

The exclusion criteria were as follows: (1) duplicate publications or studies with overlapping data; (2) studies lacking complete data or full-text access; and (3) non-English or non-Chinese publications.

### 2.3 Study selection and data extraction

Two researchers trained in systematic review methods independently screened literature using EndNote 20, performed duplicate removal and study selection, extracted data, and cross-verified findings. In cases of disagreement, a third researcher assisted in reaching a consensus.

Data were extracted from the included studies by two independent investigators. The following information was recorded: first author's name, year of publication, study location, survey period, study type, source of study subjects, mean age, sample size, sample characteristics related to oral frailty assessment and analyzed factors.

### 2.4 Quality assessment

Two researchers independently assessed the included studies and cross-checked their evaluations. In case of disagreement, a third researcher assisted in the decision-making process. All studies included in this review were cross-sectional in design. The quality of the included studies was assessed using the Joanna Briggs Institute (JBI) literature quality assessment tools (8). The JBI critical appraisal tool for cross-sectional studies comprises eight items with response options including “yes,” “no,” “unclear,” and “not applicable.” Overall quality ratings were categorized as “included,” “excluded” and “seek further info.”

### 2.5 Data analysis

Statistical analysis was performed using Stata 18.0 software. Prior to meta-analysis, data were converted for factors involving both dichotomous variables and continuous or ordinal variables. In accordance with the Cochrane Handbook guidelines for data transformation methods, continuous variables were converted into dichotomous variables. The odds ratio (OR) was used as the effect measure, with the 95% confidence interval (CI) serving as the effect analysis statistic. Heterogeneity testing indicated *I*^2^ ≤ 50%, suggesting low heterogeneity; a fixed-effects model was used for meta-analysis. If *I*^2^ > 50%, a random-effects model was used. Subgroup analyses were conducted to explore sources of heterogeneity, supplemented by sensitivity analyses. Publication bias was assessed using funnel plots and Egger's test. *P* < 0.05 was considered statistically significant.

## 3 Results

### 3.1 Study process

**Figure 1:**
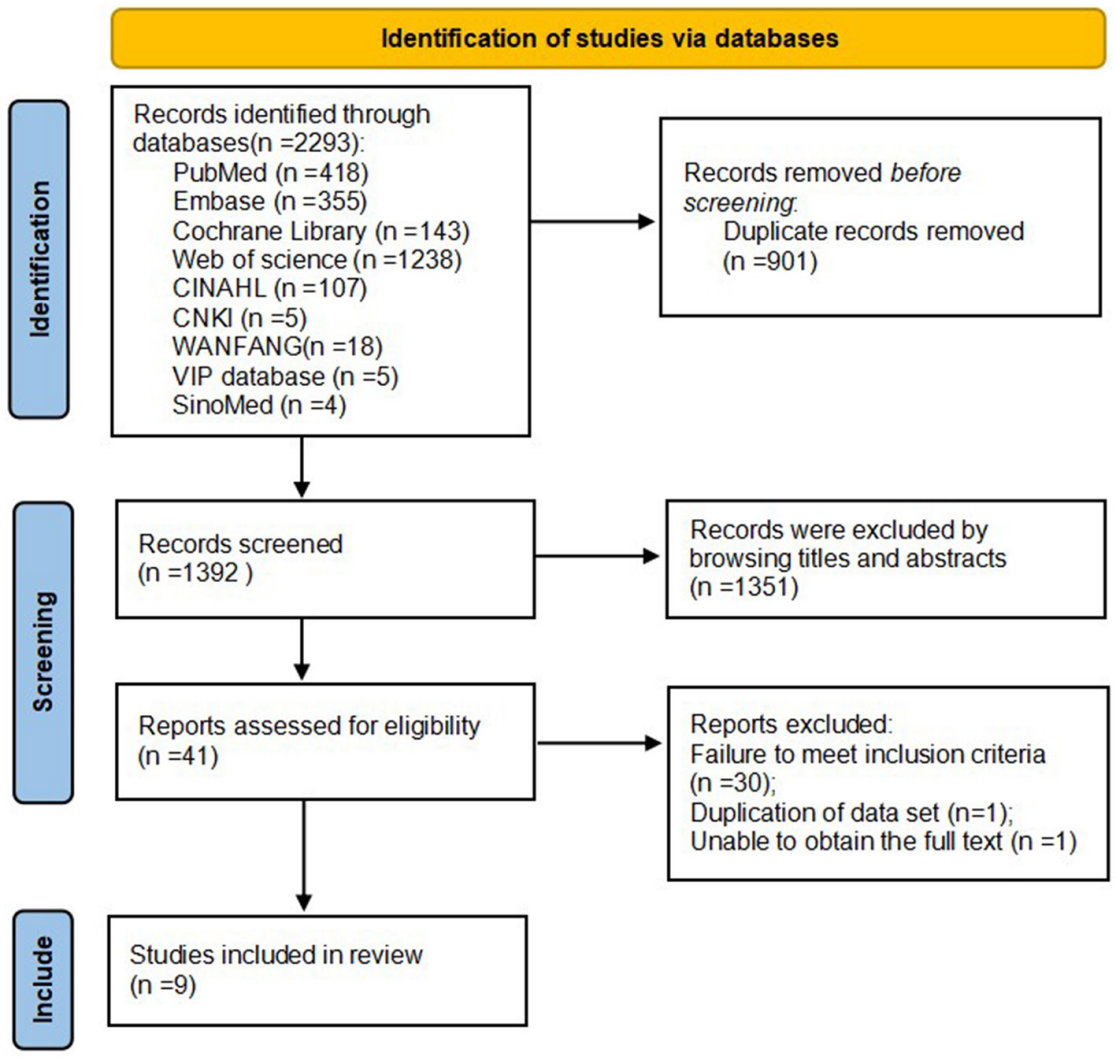
Literature screening process.

The initial search identified 2,293 records, of which nine studies met the inclusion criteria (9–17): five Chinese-language articles (10, 11, 13, 14, 16), and four English-language articles (9, 12, 15, 17). The literature screening process is shown in Figure 1.

### 3.2 Characteristics of the included studies

Based on the JBI critical appraisal tool, the overall methodological quality was moderate to high. Of the nine studies, three achieved a perfect score of 100%, four scored 87.5%, one scored 75%, and only one scored 62.5% (See Supplementary material S1). This result demonstrated that the majority of the included studies were rated as high-quality studies, and all studies were included. This study included nine cross-sectional studies, all of which used the OFI-8 scale to assess oral frailty, comprising a total of 2,395 participants.

### 3.3 Prevalence of oral frailty in older adults with diabetes

This study conducted a meta-analysis of the nine studies ultimately included, covering a total of 2,395 participants, to estimate the pooled prevalence of oral frailty among older diabetic populations. Basic characteristics of included studies are shown in Table 1. Results showed significant variation in the reported prevalence of oral frailty across the nine included studies, with reported prevalence rates ranging from 32.95 to 74.41%. Heterogeneity assessment revealed substantial heterogeneity (*I*^2^ = 95.15%, exceeding the 50% threshold) and Cochrane's *Q* test *P* < 0.01, indicating substantial heterogeneity among studies. Meta-regression analysis of oral frailty prevalence in older adults with diabetes mellitus demonstrated that sample size (*z* = −1.49, *P* = 0.18) explained 14.68% of the observed heterogeneity. Although this proportion of explained heterogeneity was relatively modest and statistically non-significant, sample size was the most influential factor among all covariates examined. Subgroup analysis revealed a lower prevalence of oral frailty in older diabetic cohorts with a sample size of ≥400 cases compared to those with <400 cases; detailed results of the subgroup analyses are presented in Table 2. None of the other covariates examined in the meta-regression showed statistically significant associations with oral frailty prevalence: mean age (*z* = 0.28, *P* = 0.79), year of publication (*z* = 0.11, *P* = 0.92), study quality score (*z* = −0.11, *P* = 0.92), publication language (*z* = 0.11, *P* = 0.92), or study population source (*z* = 0.46, *P* = 0.66).

**Table 1:** Basic characteristics of included studies.

**Authors**	**Publication year**	**Country/Region**	**Survey period**	**Study type**	**Study population source**	**Average age (Year)**	**Sample size**	**Oral frailty sample size**	**Influencing factors**
Luo et al. (9)	2025	China, Sichuan Province	2024.03–2024.10	Cross-sectional	Hospital	71.44 (≥60)	431	142	①②③④⑤⑥
Shang et al. (10)	2025	China, Henan Province	2023.10–2024.05	Cross-sectional	Hospital	71.50 (≥60)	220	103	③⑤⑦⑧⑨⑩
Tang et al. (11)	2025	China, Guangxi Province	2024.08–2024.10	Cross-sectional	Community	70.19 (≥60)	235	170	⑪⑫⑬⑭
Tian et al. (12)	2025	China, Shanxi Province	2024.06–2024.10	Cross-sectional	Hospital	68.99 (≥60)	464	213	𤘠④⑪⑫⑮⑯⑰
Yang et al. (13)	2025	China, Sichuan Province	2024.01–2024.07	Cross-sectional	Hospital	– (≥60)	207	95	①③⑤⑥⑮⑱⑲
Yi et al. (14)	2025	China, Jiangsu Province	2023.10–2025.05	Cross-sectional	Hospital	69.72 (≥60)	232	121	①⑥⑪⑱⑳㉑
Yu et al. (15)	2025	China	2023.10–2024.03	Cross-sectional	Hospital	71.2 (≥60)	211	157	㉑㉒㉓㉔
Zhong et al. (16)	2024	China, Anhui Province	2023.10–2024.02	Cross-sectional	Hospital	61.37 (≥60)	284(Build a prototype)	129	①⑮㉑㉖㉗㉘
Ishii et al. (17)	2022	Japan, Shizuoka	2019.12–2020.03	Cross-sectional	Hospital	79.7 (≥75)	111	59	⑪

①is age; ②is dysphagia; ③is HbA1c level; ④is oral health status (OHAT); ⑤number of remaining teeth; ⑥is oral health-related self-efficacy (GSEOH); ⑦is dry mouth; ⑧is nutritional risk Nutrition Risk Screening 2002 (NRS 2002) is a scale developed by Kondrup et al. to screen patients for nutritional risk. It covers three dimensions: disease severity, nutritional status indicators, and age. The total score ranges from 0 to 7; a score of ≥3 indicates the presence of nutritional risk (52); ⑨is oral health literacy (HeLD-14); ⑩is iabetes distress scale (DDS); ⑪is diabetes duration; ⑫is polypharmacy; ⑬is medication adherence; ⑭is social frailty (SFI); ⑮is smoking history; ⑯is weekly exercise frequency; ⑰is frailty status (FRAIL); ⑱is educational level; ⑲is type of chronic disease; ⑳is anxiety (GAD-7); ㉑is monthly household income per capita; ㉒is FNT; ㉓is dental restoration status; ㉔is FBG level; ㉕is cognitive impairment; ㉖is ASMI; ㉗is BMI; ㉘is difficulty chewing.

**Table 2:** Subgroup analysis results of oral frailty incidence among older diabetes patients.

**Subgroups**	**Number of include studies**	**Heterogeneity test**		**Effect model**	**Meta-analysis results**	
		*I*^2^ **(%)**	***P-*** **value**		**Incidence rate**	**95% CI**
**Sample size**						
≥400	2 (9, 12)	<0.01	<0.01	Fixed	0.40	0.36–0.43
<400	7 (10, 11, 13–17)	93.29	<0.01	Random	0.56	0.46–0.66

**Figure 2:**
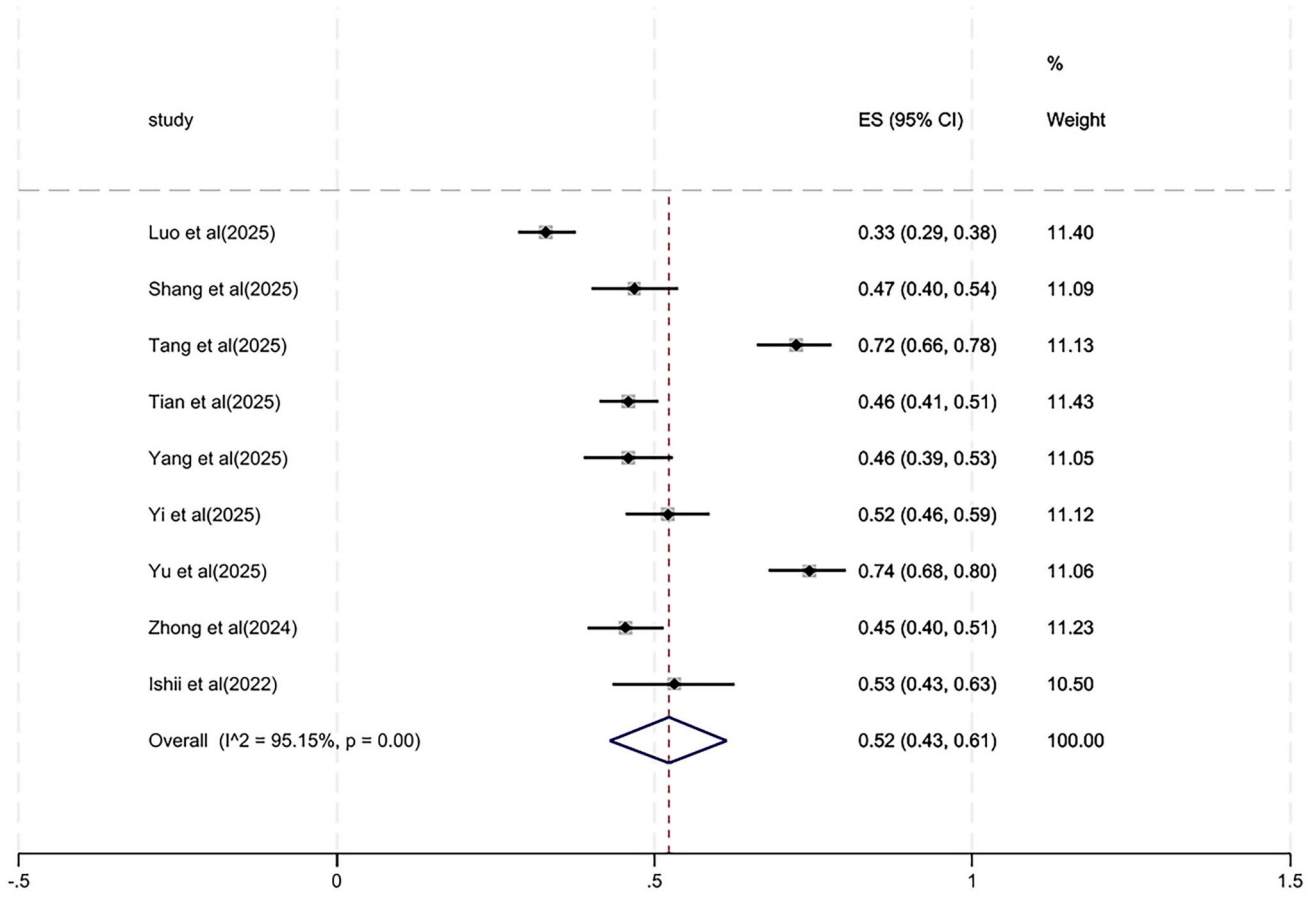
Forest plot of oral frailty prevalence among older diabetes patients.

Thus, a random-effects model was employed for analysis. The pooled prevalence estimate was 52% (95% CI: 0.43–0.61, *z* = 16.47, *P* < 0.01), indicating a 52% prevalence of oral frailty among older adults with diabetes. Figure 2 presents the forest plot.

### 3.4 Influencing factors

A meta-analysis was conducted on factors reported in at least two studies, initially identifying 10 variables eligible for inclusion. Some variables in the included studies exhibited insufficient data completeness, which precluded conversion into odds ratios (OR) or other combinable effect measures. These variables were excluded as they failed to meet the requirements for effect size pooling in meta-analysis, resulting in seven variables ultimately included for analysis. Meta-analysis results indicate that older age, smoking, polypharmacy, fewer remaining teeth, longer duration of diabetes, elevated glycated hemoglobin (HbA1c) levels, and low oral health-related self-efficacy were identified as risk factors for oral frailty in older adults with diabetes.

Five studies (9, 12–14, 16) indicated that age is a factor influencing oral frailty in older adults with diabetes. Two studies (12, 16) were excluded due to insufficient data to derive OR or other effect sizes. Ultimately, three studies (9, 13, 14) were included in the analysis. No significant heterogeneity was observed (*I*^2^ = 36.4%, *P* = 0.208). Results from the fixed-effects model indicated that older age is a risk factor for oral frailty in older adults with diabetes (OR = 3.91, 95% CI: 2.13–5.68, *P* < 0.01).

Two studies (12, 16) examined the impact of smoking on oral frailty in older adults with diabetes. Homogeneity was observed among the studies (*I*^2^ <0.1%, *P* = 0.553). Results from the fixed-effects model indicated that smoking behavior constitutes a risk factor for oral frailty in older adults with diabetes (OR = 4.64, 95% CI: 1.95–7.33, *P* < 0.01). Two studies (11, 12) examined the effect of polypharmacy on oral frailty in older adults with diabetes. Moderate heterogeneity existed between studies (*I*^2^ = 9.7%, *P* = 0.293). The fixed-effects model showed polypharmacy as a risk factor for oral frailty in older adults with diabetes (OR = 8.70, 95% CI: 3.26–14.14, *P* < 0.01).

Five studies (11–14, 17) indicated that diabetes duration is a factor influencing oral frailty in older adults with diabetes. Two studies (11, 17) provided insufficient data to derive OR or other effect sizes. One study (13) included a categorical variable with significance in only one group and was thus excluded. Ultimately, two studies (12, 14) were included in the analysis. Low heterogeneity was observed among the included studies (*I*^2^ = 24.3%, *P* = 0.250). The fixed-effects model revealed that longer diabetes duration is a risk factor for oral frailty in older adults with diabetes (OR = 3.29, 95% CI: 1.55–5.04, *P* < 0.01). Three studies (9, 10, 13) examined the impact of HbA1c on oral frailty in older adults with diabetes. Homogeneity was observed among these studies (*I*^2^ <0.1%, *P* = 0.996). Fixed-effects model results indicated that elevated HbA1c levels (≥7%) constitute a risk factor for oral frailty in older adults with diabetes (OR = 3.61, 95% CI: 1.47–5.74, *P* < 0.01).

Four studies (9, 10, 13, 15) indicated that the number of remaining teeth is a factor influencing oral frailty in older adults with diabetes. Two studies (13, 15) were excluded due to insufficient data to derive OR or other effect sizes. Ultimately, two studies (9, 10) were included in the analysis. Low heterogeneity was observed among the included studies (*I*^2^ = 8.4%, *P* = 0.296). The fixed-effects model revealed that having fewer than 20 remaining teeth was a risk factor for oral frailty in older adults with diabetes (OR = 11.84, 95% CI: 4.47–19.22, *P* < 0.01). Two studies (9, 14) examined the impact of oral health-related self-efficacy [assessed using the GSEOH scale (18)] on oral frailty in older adults with diabetes. Moderate heterogeneity existed between studies (*I*^2^ = 31.9%, *P* = 0.226). Fixed-effects model results indicated that low oral health-related self-efficacy (GSEOH scale score <50) was a risk factor for oral frailty in older adults with diabetes (OR = 0.18, 95% CI: 0.10–0.27, *P* = 0.001).

### 3.5 Sensitivity and publication bias analysis

**Figure 3:**
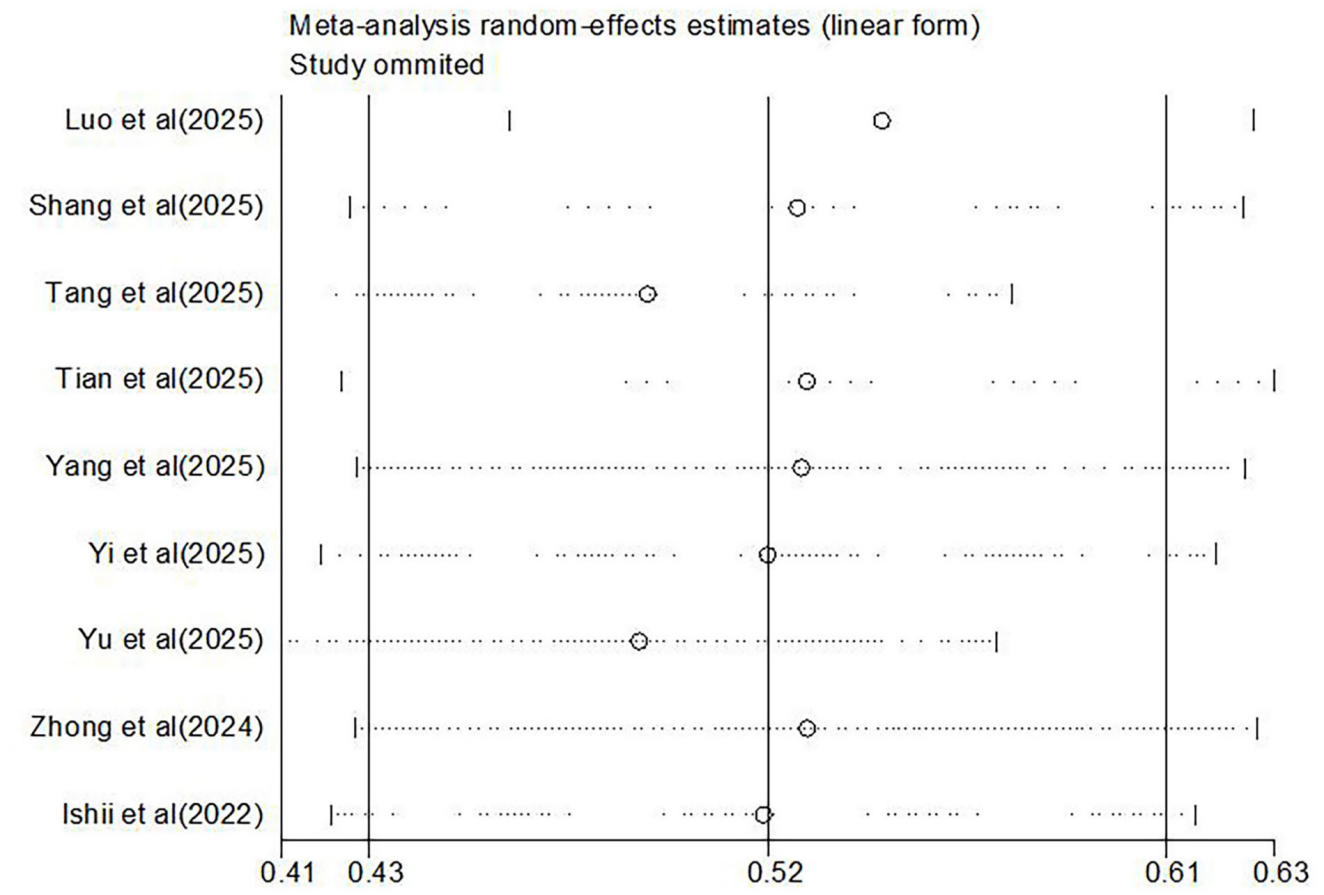
Sensitivity analysis of oral frailty incidence in older diabetes patients.

**Figure 4:**
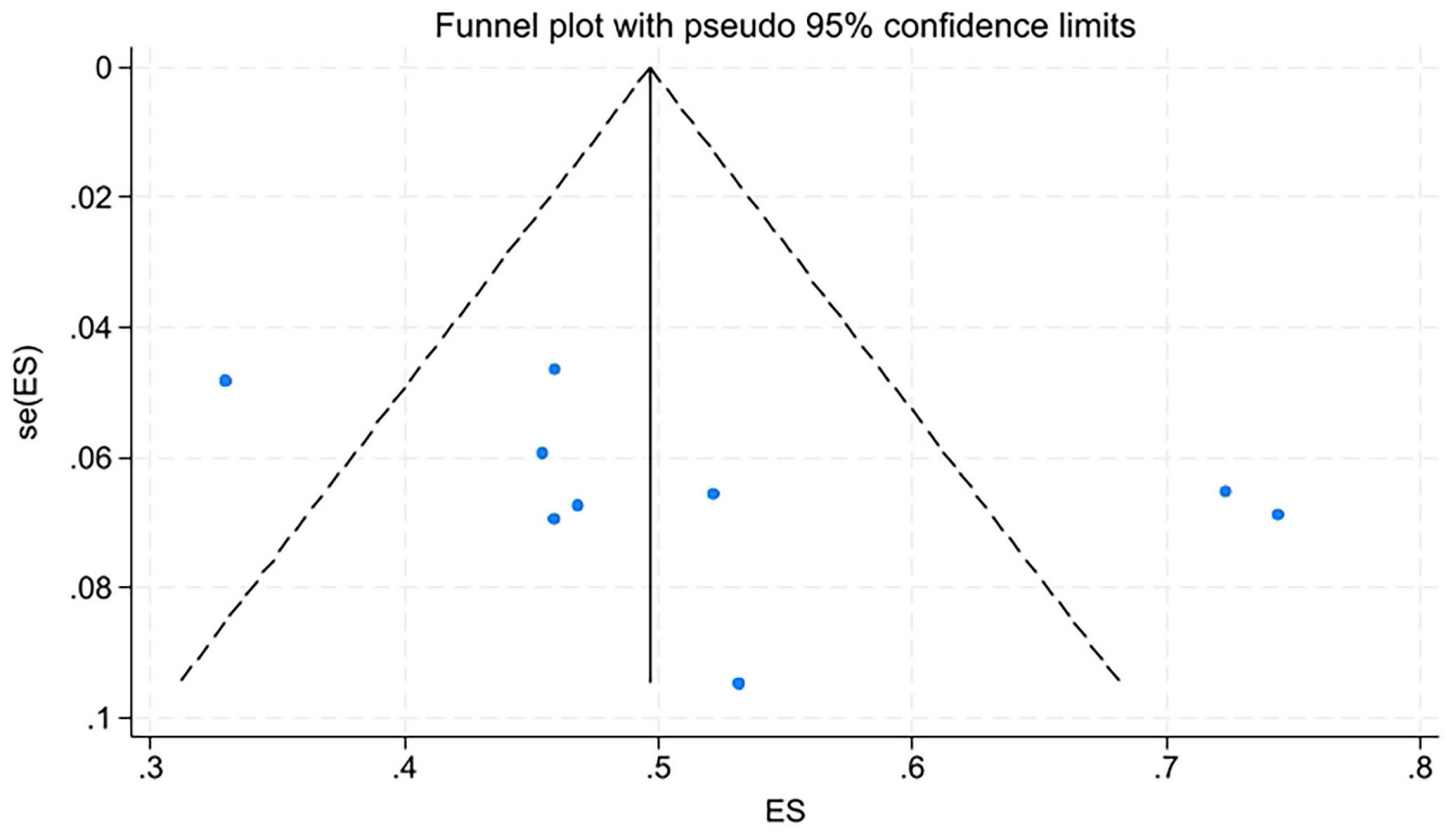
Publication bias analysis of oral frailty incidence in older diabetes patients.

Removing any individual study from the sensitivity analysis did not significantly alter the pooled prevalence rate of oral frailty among older adults with diabetes, indicating robust and stable results of the meta-analysis results. Furthermore, funnel plot analysis and Egger's test (*t* = 1.59, *P* = 0.155 > 0.05) indicated no publication bias among the included studies. See Figures 3, 4. Sensitivity analysis was performed on the included influencing factors by altering the effect model. After switching the effect models, the pooled effect sizes and directions remained consistent, suggesting the robustness of the findings. Egger's tests for these factors showed no evidence of publication bias (Table 3).

**Table 3:** Sensitivity and publication bias analysis results of factors affecting oral frailty in older diabetes patients.

**Influence factors**	**Merge analysis results**		**Merge analysis results after model transformation**		**Egger's (95% CI)**
	**Effects model**	**OR (95% CI)**	**Effects model**	**OR (95% CI)**	
Age	Fixed	3.91 (2.13–5.68)	Random	4.54 (1.84–7.25)	0.951 (−109.111, 110.436)
Smoking	Fixed	4.64 (1.95–7.33)	Random	4.64 (1.95–7.33)	NA
Polypharmacy	Fixed	8.70 (3.26–14.14)	Random	8.63 (2.88–14.38)	NA
Duration of diabetes	Fixed	3.29 (1.55–5.04)	Random	3.50 (1.21–5.80)	NA
HbA1c level	Fixed	3.61 (1.47–5.74)	Random	3.61 (1.47–5.74)	0.050 (−0.001, 0.454)
Number of remaining teeth	Fixed	11.84 (4.47–19.22)	Random	12.03 (4.13–19.92)	NA
Oral health-related self-efficacy	Fixed	0.18 (0.10–0.27)	Random	0.20 (0.08–0.32)	NA

NA, Insufficient number for Egger's test.

## 4 Discussion

The results of the meta-analysis in this study demonstrate that the prevalence of oral frailty among older patients with diabetes mellitus was 52%, with significant heterogeneity. To explore the sources of heterogeneity, we conducted meta-regression analysis and subgroup analysis. However, the sources of heterogeneity remained unresolved; therefore, a random-effects model was employed in the final pooled prevalence analysis. Subgroup analysis by sample size revealed higher oral frailty rates in studies with fewer than 400 participants, consistent with previous findings (19). Studies with smaller sample sizes typically have higher risks of publication bias and selection bias, potentially leading to more extreme prevalence estimates. Additionally, smaller sample sizes reduce the statistical power to detect differences between groups (20, 21), resulting in an overestimation of the investigated oral frailty prevalence.

Systematic reviews estimate that the overall prevalence of oral frailty in the general older population is approximately 30% (19, 22–24). Diabetes mellitus and its complications are established risk factors for oral diseases (5, 6). Chronic hyperglycemia in diabetic patients damages oral tissues, Combined with pathological changes such as abnormal neutrophil function and microangiopathy (5, 6, 25, 26). Additionally, pathophysiological alterations induced by diabetes, such as insufficient blood perfusion and immune suppression, further increase the risk of oral diseases (6, 27, 28). Insulin resistance and chronic inflammation in diabetes impair muscle strength and motor function (29), elevating the risk of oral frailty. In addition to these pathophysiological pathways, older diabetic patients exhibit a high prevalence of social frailty (30). Reduced social interaction leads to neglect of oral hygiene, accelerating the progression of oral frailty (31). Owing to the confluence of these factors, the prevalence of oral frailty in older diabetic patients is higher than in non-diabetic older individuals.

The findings of this study indicate that age is associated with the prevalence of oral frailty in older patients with diabetes mellitus: older age was associated with higher prevalence of oral frailty. Age is recognized as a significant factor influencing oral health in diabetic patients, with poorer oral health and higher oral frailty probability associated with advancing age (32, 33). From a biological perspective, first, older patients exhibit elevated levels of the senescence-associated secretory phenotype (SASP). SASP not only exacerbates chronic inflammatory responses but also accelerates the aging process of both normal and immune cells, thereby compromising normal cellular function (34) and leading to deteriorating oral health. Second, with advancing age, oral physiological parameters—including dentition, periodontal tissues, oral mucosa, salivary gland function, and masticatory function—all demonstrate an age-related decline (35), which directly contributes to poorer oral health. From a systemic perspective, oral health is closely linked to geriatric syndromes (34). With increasing age, the prevalence of malnutrition, sarcopenia, and frailty rises among the older adults, and these geriatric syndromes further exacerbate oral health deterioration. Additionally, older diabetic patients often have a longer duration of diabetes, which further increases the risk of oral frailty. This highlights the importance of healthcare providers to recognize the impact of age on oral frailty in older adults with diabetes. Future practice and research should implement age-stratified oral frailty screening while concurrently monitoring both oral and systemic health in this population, thereby providing evidence for developing targeted intervention strategies.

The results reveal that smoking or polypharmacy is associated with the prevalence of oral frailty in older diabetic patients; individuals who smoke or take multiple medications have a higher prevalence of oral frailty. Smoking triggers systemic inflammatory responses, reduces tissue perfusion blood flow, impairs immune system function, and disrupts tissue repair processes (36, 37), thereby exacerbating the onset and progression of oral diseases and increasing susceptibility to oral frailty symptoms. In addition, studies indicate that longer smoking cessation duration correlates with more substantial recovery and improvement in oral health (38). Thus, healthcare providers should educate older adults with diabetes about the harms of smoking to both oral and overall health, support smoking cessation efforts and develop and optimize evidence-based strategies to promote smoking cessation in this population. Research confirms that individuals with polypharmacy generally exhibit poorer oral function (39). A potential mechanism involves certain medications commonly used to treat chronic diseases, which may induce xerostomia and suppress the swallowing reflex (40). This disrupts the oral environment, thereby increasing the risk of oral frailty. Older adults with diabetes frequently have multiple comorbidities, making polypharmacy a common occurrence (41). Healthcare providers should enhance oral health monitoring and intervention for patients taking medications that may affect oral function or those with polypharmacy, remaining vigilant for the onset of oral frailty.

This study demonstrates that diabetes duration or glycated hemoglobin (HbA1c) levels are associated with the incidence of oral frailty in older diabetic patients. Those with a longer diabetes duration or HbA1c ≥7% exhibit a higher prevalence of oral frailty. Diabetes itself represents a risk factor for oral health. As diabetes progresses, patients experience changes such as restricted blood flow, suppressed immune function, and impaired cellular renewal (42). These alterations increase the risk of oral diseases (27, 28), with more severe oral conditions associated with longer disease duration (43). Daily interdental cleaning is a crucial oral hygiene practice for maintaining gingival health (44). Teaching diabetic patients standardized oral self-care techniques, such as proper brushing and flossing, is a key component of comprehensive diabetes self-management. HbA1c is a key indicator for assessing long-term glycemic control. Persistent hyperglycemia leads to immune dysfunction, chronic inflammation, and disruption of the oral microbiome (45). Concurrently, patients with poor glycemic control face heightened risks of complications (46), such as vascular and neuropathic disorders, which further compromise oral health and increase the likelihood of oral frailty. Thus, healthcare providers should prioritize monitoring glycemic control in older adults with diabetes with existing oral frailty, providing professional guidance to facilitate scientifically sound and effective blood glucose management.

Our findings show that the number of remaining teeth or oral health-related self-efficacy is associated with the prevalence of oral frailty in older diabetic patients. Individuals with fewer than 20 remaining teeth or low oral health-related self-efficacy (GSEOH scale score <50) have a higher prevalence of oral frailty. Having fewer than 20 natural teeth directly impairs the functional integrity of oral function in older adults with diabetes, leading to reduced masticatory function. To compensate for impaired chewing, patients often consume large-particle-size foods to meet basic nutritional needs (47). However, since older adults have reduced digestive function, and ingesting large particles hinders the digestion, absorption, and metabolism of nutrients. Oral frailty is closely associated with malnutrition (48). Impaired nutrient absorption further exacerbates oral health deterioration, creating a vicious cycle that increases the risk of oral frailty. Assisting older adults with diabetes with dental restoration or denture fitting not only directly improves chewing function, nutrition, and oral health but also enhances quality of life and mental wellbeing (49). Oral health-related self-efficacy is closely associated with oral health. Self-efficacy serves as a predictor of individual health behavior change, while the onset and progression of oral diseases are mediated by individual health behaviors (50, 51). When older adults with diabetes exhibit low oral health-related self-efficacy, their motivation and capacity to perform in proactive oral health behaviors diminish, thereby increasing the risk of oral frailty. Research has confirmed that appropriately adjusting an individual's oral health behaviors can effectively alter the disease progression of oral diseases (18). Therefore, healthcare providers should implement targeted oral health promotion programs for older adults with diabetes. Through health education and skill guidance, these programs should enhance their awareness of oral health and self-efficacy, foster positive oral health behaviors, improve oral health status, and reduce the risk of oral frailty.

## 5 Conclusion

In summary, this study identifies age, smoking, polypharmacy, diabetes duration, HbA1c levels, number of remaining teeth, and oral health-related self-efficacy as factors associated with the incidence of oral frailty in older patients with diabetes mellitus. Based on these findings, clinicians should incorporate oral frailty assessment into routine monitoring for older adults with diabetes. Based on individual patient profiles and clinical contexts, and through multidisciplinary collaboration, they should develop comprehensive and personalized intervention plans to improve or delay the progression of oral frailty, thereby maintaining patients' oral health and enhancing their quality of life.

## 6 Limitations

We recognize that this meta-analysis has limitations. First, all included studies adopted a cross-sectional design, which precludes causal inference between the identified factors and oral frailty. Second, significant heterogeneity was observed in the pooled prevalence estimates. Despite conducting meta-regression and subgroup analyses to explore potential sources, the majority of heterogeneity remains unexplained, potentially originating from unmeasured clinical or methodological differences across studies. Third, the generalizability of our findings is limited to Asian populations. Future research should incorporate participants from more diverse ethnicities and geographical regions. Fourth, the assessment of oral frailty in the included studies relied solely on the OFI-8 scale, which might not fully align with definitions used in other assessment tools; therefore, our results should be interpreted specifically within the context of the OFI-8 criteria. Finally, the investigation of factors such as polypharmacy was limited by the available data, which primarily defined exposure based on the number of medications rather than their specific classes or pharmacological effects, thereby hindering the identification of drug-specific risks.

## Data Availability

The original contributions presented in the study are included in the article/Supplementary material, further inquiries can be directed to the corresponding author.
