# Supplementary material for: Prevalence and influencing factors of oral frailty in older adults with diabetes: a systematic review and meta-analysis
Source: Front Public Health. 2025 Nov 28;13:1702509. doi: 10.3389/fpubh.2025.1702509 (PMC12698406; doi:10.3389/fpubh.2025.1702509)
Supplement: Supplementary file 1 [file Supplementary_file_1.docx]

Supplementary Material

**Search strategy**

**PubMed**

((("Aged"[MeSH Terms] OR "older people"[Title/Abstract] OR "older adult*"[Title/Abstract] OR "elderly"[Title/Abstract] OR "geriatric"[Title/Abstract] OR "senior" [Title/Abstract]) AND ("Diabetes Mellitus"[MeSH Terms] OR "Diabetes Mellitus, Type 2"[MeSH Terms] OR "Diabetes Mellitus, Type 1"[MeSH Terms] OR "diabetes"[Title/Abstract] OR "diabetic"[Title/Abstract] OR "DM"[Title/Abstract])) AND ("oral frailty"[Title/Abstract] OR "oral frail"[Title/Abstract] OR "oral weakness"[Title/Abstract] OR "Oral health"[Title/Abstract] OR "Oral function"[Title/Abstract])) AND ("impact*"[Title/Abstract] OR "cause*"[Title/Abstract] OR "reason*"[Title/Abstract] OR "association"[Title/Abstract] OR "relationship"[Title/Abstract] OR "effect*"[Title/Abstract]) Sort by: Publication Date

**Web of Science**

"Aged" OR "older people" OR "older adult*" OR "elderly" OR “geriatric” OR “senior” (Topic) and "Diabetes Mellitus" OR "Diabetes Mellitus, Type 2" OR "Diabetes Mellitus, Type 1" OR "diabetes" OR "diabetic" OR "DM" (Topic) and "oral frailty" OR "oral frail" OR “oral weakness” OR “Oral health” OR “Oral function” (Topic) and "impact*" OR "cause*" OR "reason*" OR "association" OR "relationship" OR "effect*" (Topic) and Preprint Citation Index (Exclude – Database)

**CINAHL**

XB ("Aged" OR "older people" OR "older adult*" OR "elderly" OR “geriatric” OR “senior”) AND XB ("Diabetes Mellitus" OR "Diabetes Mellitus, Type 2" OR "Diabetes Mellitus, Type 1" OR "diabetes" OR "diabetic" OR "DM") AND XB ("oral frailty" OR "oral frail" OR “oral weakness” OR “Oral health” OR “Oral function”) AND XB ("impact*" OR "cause*" OR "reason*" OR "association" OR "relationship" OR "effect*")

**Embase**

("Aged" OR "older people" OR "older adult*" OR "elderly" OR “geriatric” OR “senior”:ab,ti) AND ("Diabetes Mellitus" OR "Diabetes Mellitus, Type 2" OR "Diabetes Mellitus, Type 1" OR "diabetes" OR "diabetic" OR "DM":ab,ti) AND ("oral frailty" OR "oral frail" OR “oral weakness” OR “Oral health” OR “Oral function”) AND ("impact*" OR "cause*" OR "reason*" OR "association" OR "relationship" OR "effect*")

**Cochrane Library**

"Aged" OR "older people" OR "older adult*" OR "elderly" OR “geriatric” OR “senior” in Title Abstract Keyword AND "Diabetes Mellitus" OR "Diabetes Mellitus, Type 2" OR "Diabetes Mellitus, Type 1" OR "diabetes" OR "diabetic" OR "DM" in Title Abstract Keyword AND "oral frailty" OR "oral frail" OR “oral weakness” OR “Oral health” OR “Oral function” in Title Abstract Keyword AND "impact*" OR "cause*" OR "reason*" OR "association" OR "relationship" OR "effect*" in Title Abstract Keyword - (Word variations have been searched)

**CINAHL/CNKI/WANFANG/VIP Database/SinoMed**

(AB=diabetes) AND (oral frailty)

**Quality assessment**

The quality assessment appraisal was performed by two independent assessors using the standardized Joanna Briggs Institute (JBI) critical appraisal tool prepared for cross-sectional studies and cohort studies. The tools have ‘Yes’, ‘No’, ‘Unclear’ or ‘not applicable’ responses, and scores were given 1 for ‘Yes’, 0 for ‘No’ and ‘Unclear’ responses. Scores for each item were summed up and transformed into percentages. The average score of the two independent assessors were taken. Only studies that scored ≥50% were considered for systematic review and meta-analysis.

**JBI Critical Appraisal Checklist for Analytical cross-sectional studies:**

1. Were the criteria for inclusion in the sample clearly defined?

2. Were the study subjects and the setting described in detail?

3. Was the exposure measured in a valid and reliable way?

4. Were objective, standard criteria used for measurement of the condition?

5. Were confounding factors identified?

6. Were strategies to deal with confounding factors stated?

7. Were the outcomes measured in a valid and reliable way?

8. Was appropriate statistical analysis used?

Based on the above criteria, the quality score for analytical cross-sectional studies is given below.

| **Author** | **Criteria and corresponding scores** | | | | | | | | | **Total** | **%** | **Overall appraisal** |
| --- | --- | --- | --- | --- | --- | --- | --- | --- | --- | --- | --- | --- |
|  | **#1** | **#2** | | **#3** | **#4** | **#5** | **#6** | **#7** | **#8** |  |  |  |
| Luo et al (1) | Yes | Yes | Yes | | Yes | Yes | Yes | Yes | Yes | 8 | 100 | Include |
| Shang et al (10) | Yes | Yes | Yes | | Yes | Yes | Yes | Unclear | Yes | 7 | 87.5 | Include |
| Tang et al (11) | Yes | Yes | Yes | | Yes | Yes | Yes | Unclear | Yes | 7 | 87.5 | Include |
| Tian et al (12) | Yes | Yes | Yes | | Yes | Yes | Yes | Yes | Yes | 8 | 100 | Include |
| Yang et al (13) | Yes | Yes | Yes | | Yes | Yes | Yes | Unclear | Yes | 7 | 87.5 | Include |
| Yi et al (14) | Yes | Yes | Unclear | | Yes | Yes | Yes | Unclear | Yes | 6 | 75 | Include |
| Yu et al (15) | Yes | Yes | Yes | | Yes | Yes | Yes | Yes | Yes | 8 | 100 | Include |
| Zhong et al (16) | Yes | Yes | Unclear | | Yes | Yes | Yes | Yes | Yes | 7 | 87.5 | Include |
| Ishii et al (17) | Unclear | Yes | Unclear | | Yes | Yes | Yes | Unclear | Yes | 5 | 62.5 | Include |

## References

1. Luo W, Zhou J, Qiu L, Zhao L. Influencing factors of oral frailty in elderly patients with type 2 diabetes in China: a cross-sectional study based on the integral model of frailty. BMC Oral Health. (2025) 25:546. doi: 10.1186/s12903-025-05815-8
2. Shang XH, Du YF, Wen BL, Jia QM, Zheng Y, Hu YN, Li LM. Current status of oral frailty and its influencing factors in elderly diabetic patients. Chin J Mod Nurs. (2025) 31: 1925–1930. doi: 10.3760/cma.j.cn115682-20240723-04121
3. Tang L, Yang J, Zhang YH, Wang YN, Wei SJ, Jin HQ, Mo YD. Correlation between oral frailty and social frailty in community-dwelling elderly patients with type 2 diabetes. Chin Gen Pract Nurs. (2025) 23: 2397–2402.
4. Tian C, Li N, Gao Y, Yan Y. Analysis of the current status and influencing factors of oral frailty in elderly patients with type 2 diabetes mellitus in Taiyuan, China. BMC Geriatr. (2025) 25:416. doi: 10.1186/s12877-025-06052-y
5. Yang WJ, Fang M, Wang SY. Current status of oral frailty and analysis of its influencing factors in elderly patients with type 2 diabetes. Chin Nurs Manag. (2025) 25: 363–368.
6. Yi H, Wu J, Chen XY, Hong KX, Chen ZP. Investigation on the current status of oral frailty and analysis of its influencing factors in elderly diabetic patients. Pract Geriatr. (2025) 39: 723–726, 731.
7. Yu J, Ye A, Fei Y, Wang D, Zhang Y, Li X. Associated factors of oral frailty in older adults with long-term T2DM duration of more than 10 years. BMC Geriatr. (2025) 25:259. doi: 10.1186/s12877-025-05925-6
8. Zhong L, Zhang H, Xu J, Lu YW, Xiang XT. Construction of a nomogram prediction model for the risk of oral frailty in elderly patients with type 2 diabetes. J Clin Med Pract. (2024) 28(16): 98–103, 108.
9. Ishii M, Yamaguchi Y, Hamaya H, Iwata Y, Takada K, Ogawa S, Imura M, Akishita M. Influence of oral health on frailty in patients with type 2 diabetes aged 75 years or older. BMC Geriatrics. (2022) 22:145. doi: 10.1186/s12877-022-02841-x
